# Effects of Lactic Acid and Glyceryl Lactate on Growth Performance, Antioxidant Capacity, and Intestinal Health of Piglets

**DOI:** 10.3390/antiox14040391

**Published:** 2025-03-26

**Authors:** Shuaiju Guo, Huiling Chu, Bangwang Peng, Junlong Niu, Xiaopeng Yang, Yongpeng Guo, Zhixiang Wang, Wei Zhang

**Affiliations:** 1 College of Animal Science and Technology, Henan Agricultural University, Zhengzhou 450046, China; guoshuaiju@stu.henau.edu.cn (S.G.); pengbangwang@stu.henau.edu.cn (B.P.); niujunlong@stu.henau.edu.cn (J.N.); yxp@stu.henau.edu.cn (X.Y.); guoyp@henau.edu.cn (Y.G.); 2 College of Food and Information Engineering, Zhengzhou University of Light Industry, Zhengzhou 450000, China; chuhuiling@jindanlactic.com

**Keywords:** lactic acid, glyceryl lactate, growth performance, antioxidant capacity, SCFAs, intestinal health

## Abstract

The aim of this study was to evaluate the effects of lactic acid and glyceryl lactate on growth performance, antioxidant capacity, and intestinal health in piglets. This study included 240 castrated male piglets (initial body weight: 7.50 ± 0.54 kg) assigned to four groups: CON (basal diet), LA (basal diet + 0.5% lactic acid), GL (basal diet + 0.5% glyceryl lactate), and LG (basal diet + 0.5% lactic acid + 0.5% glyceryl lactate). Each group had six replicates of 10 piglets. The trial lasted 28 days. Compared with the control group, the GL and LG groups showed enhanced growth performance and reduced diarrhea rate in piglets. The LA and LG groups showed decreased intestinal chyme pH and increased digestive enzyme activities. Moreover, the GL and LG groups displayed elevated jejunal mRNA levels of the tight junction protein *occludin* and mucin *MUC2*, enhanced expression levels of *Nrf2* signaling pathway genes, increased activities of the antioxidant enzymes GPX and CAT, and reduced MDA content. Acidifier supplementation also modulated cecal bacterial abundance and short-chain fatty acid (SCFA) content. Genera such as *Faecalibaculum*, *Nocardiopsis*, *Collinsella*, *CAG269*, *Allobaculum*, and *Enterococcus* were affected. In conclusion, glyceryl lactate and its combination with lactic acid improved piglet growth performance by enhancing intestinal barrier function, antioxidant capacity, microbial community structure, and SCFA production.

## 1. Introduction

In the contemporary high-density cultivation industry, segregated early weaning is often implemented to enhance the reproductive capacity of sows and mitigate the spread of diseases, thereby increasing the output and financial profitability of swine production [1]. However, the physiological and psychological responses triggered by isolation and environmental changes during the weaning period can result in early weaning stress syndrome. This condition frequently presents as abnormal intestinal morphology and function, decreased digestion and absorption, compromised intestinal barriers, and a range of issues including reduced feed intake and increased diarrhea [2]. These deleterious symptoms are associated with inflammatory responses, oxidative stress, and imbalances in intestinal microflora, making the development of effective strategies to improve the health status of weaned piglets critical.

Traditionally, antibiotics have been successfully utilized to alleviate weaning stress and enhance performance [3]. Nevertheless, the excessive application of antibiotics can result in high levels of residue, potentially fostering the emergence of antibiotic resistance in animals and humans alike, thereby endangering human health [4]. With the global ban on antibiotics, various alternatives have been explored to replace antibiotics in animal production while maintaining performance and animal health. Among these alternatives, acidifiers are regarded as one of the most promising options for replacing antibiotics in the diets of weaned piglets.

The incorporation of feed acidifiers can lower the pH in the gastrointestinal tract, enhance digestive enzyme activity, inhibit the growth of harmful bacteria, reduce the frequency of diarrhea, and improve the growth performance of animals [5,6]. Lactic acid, an organic acid, effectively reduces the pH in the stomach and foregut, enhances digestion and absorption processes, and inhibits the proliferation of key microbial pathogens [7,8]. Glyceryl lactate, classified as a short-chain fatty acid (SCFA) ester, decomposes gradually into lactic acid and glycerol within the gastrointestinal tract. This decomposition provides a substantial amount of lactic acid to the hindgut, which is advantageous for enhancing antioxidant capacity and energy metabolism in animals [9,10].

We hypothesize that lactic acid and glyceryl lactate can jointly maintain gastric and overall intestinal pH levels to improve intestinal health issues in weaned piglets. Accordingly, the aim of this study is to systematically evaluate the effects of lactic acid and glyceryl lactate on the growth performance, antioxidant capacity, and intestinal health of piglets.

## 2. Materials and Methods

### 2.1. Animal Ethics Statement

All experimental protocols in this study were reviewed and approved by the Animal Ethics Committee of Henan Agricultural University (protocol code HNND2024031234).

### 2.2. Experimental Animals and Experimental Design

Lactic acid and glyceryl lactate were procured from Henan Jindan Lactic Acid Technology Co., Ltd. (Zhoukou, China). Two hundred and forty healthy, 21-day-old piglets (Duroc × Landrace × Large White) with an initial body weight of (7.50 ± 0.54 kg) were selected for the trial immediately after weaning. The piglets were randomly divided into four treatments, with six replicates per treatment and 10 piglets per replicate. A single-factor experimental design was employed, which included the CON group (basal diet), LA group (basal diet with 0.5% lactic acid), GL group (basal diet with 0.5% glyceryl lactate), and LG group (basal diet with 0.5% lactic acid and 0.5% glyceryl lactate). The trial period lasted for 28 days. During the trial, the piglets were provided with unlimited access to feed and water, and they underwent standard rearing and vaccination practices. The basal diet was prepared in accordance with the nutritional guidelines for weaned piglets suggested by NRC 2012 [11]. The composition and nutritional levels of the basal diet are detailed in Table S1. The gross energy, crude protein, Ca, lysine, methionine, and threonine levels in the diets were determined according to ISO 9831:1998 [12], GB/T 6432-2018 [13], GB/T 13885-2017 [14], and GB/T 18246-2019 [15], respectively [16].

During the formal experiment, acidifiers were mixed into the basal diet every morning and added in measured quantities to the feeders of each treatment group to ensure ad libitum feeding, with the amount of feed adjusted daily based on consumption. The pigpens were cleaned of excrement and urine regularly, and health conditions were monitored and documented, with any unwell pigs promptly segregated. The room temperature was maintained at approximately 25 °C, with humidity levels ranging from 65% to 70% throughout the experiment. Daily feed intake was recorded, and the piglets were monitored for diarrhea in each pen, documenting the count of affected piglets in each treatment replicate.

### 2.3. Growth Performance

The incidence of piglet diarrhea was monitored throughout the trial for each replicate, and daily feed consumption and leftovers were meticulously tracked to determine the ADG, ADFI, and feed conversion ratio (feed intake to body weight gain ratio [F:G]) for each replicate. Fecal scoring of the piglets was conducted daily (criteria are presented in Table S2), with scores of 2 or higher indicating the occurrence of diarrhea.

The diarrhea rate (%) was calculated as follows: (Number of diarrhea episodes during the trial/(Total number of piglets in the trial × Number of days of the trial)) × 100%.

### 2.4. Sampling

On day 29, a piglet (49 days old) per replicate, with weight around the mean, was chosen, administered pentobarbital sodium (50 mg/kg BW) for sedation, and subsequently euthanized. The jejunal samples were rapidly extracted. A 4 cm long portion was immersed in 4% paraformaldehyde for histological processing, while the remainder of the samples were dispensed into 2 mL vials, snap-frozen in liquid nitrogen, and stored. Samples from the cecum and jejunum were collected, with the jejunal chyme’s pH measured using a PBS-3C pH meter from Leici, China. The leftover samples were preserved in 5 mL cryogenic vials at −80 °C for subsequent analysis.

### 2.5. Digestive Enzyme Activities of Jejunum Digesta

Jejunum samples were blended in ice-chilled physiological saline at a 1:9 (*w/v*) ratio for 5 min. After blending and centrifugation for 10 min, supernatants were obtained and assayed for α-amylase, lipase, and neutral protease activities using kits from Shanghai Enzyme-Link.

### 2.6. Intestinal Morphology

Jejunum samples were taken out of the fixative, dehydrated, and then embedded in paraffin. After each jejunum sample was sectioned to a thickness of 5 μm, they were stained with hematoxylin and eosin. Representative microscopic photographs were captured using a Leica DMI3000B microscope (Leica, Wetzlar, Germany). Each section was subjected to 10 repeated measurements, and the average values were taken for statistical analysis [17].

### 2.7. Expression Level of Jejunum Gene in Piglets

The frozen jejunum samples were placed in a mortar, and liquid nitrogen was added for grinding. We weighed out 0.05 g of the ground sample into a 1.5 mL centrifuge tube and quickly added 1 mL of TRIzol reagent (TaKaRa, Beijing, China) to homogenize and extract total RNA. The sample concentration was measured using a spectrophotometer and the Nano-Drop 2000 software, calibrated with RNase-free water to zero, and 1 μL was used to determine the sample concentration. Reverse transcription was performed following the protocol from a Takara kit (Kyoto, Japan) to yield cDNA. This cDNA was diluted 10-fold using RNase-free water and stored at −20 °C for subsequent applications. Gene-specific amplicons were verified using melting curve analysis, and a 10 μL quantitative PCR (qPCR) protocol was implemented. The mRNA expression levels of the target genes were normalized using the endogenous reference gene β-actin, based on the 2^−ΔΔCt^ method. The primer sequences for the intestinal barrier and function genes (*ZO-1*, *occludin*, *claudin-1*, *MUC2*, and *JAM2*) and the antioxidant pathway genes (*Nrf2*, *HO-1*, *NQO1*, *GPX1*, *SOD1*, and *CAT*) used in q-PCR are presented in Table S3.

### 2.8. Determination of Antioxidant Indexes in Jejunum

Assessment of the antioxidant indexes in jejunal tissues was conducted following the instructions provided with the kit assay from Nanjing Jiancheng Biological Company (Nanjing, China).

### 2.9. Gut Microbes

Total DNA was isolated from intestinal contents using a DNA extraction kit according to previous studies, after which DNA quality and concentration were examined. The V3-V4a fragments of 16S rDNA were amplified using a 20 μL PCR reaction solution, with DNA from intestinal contents serving as the template. The resulting PCR products were resolved and purified, and then libraries were constructed and sequenced according to the protocols of the Illumina MiSeq platform in Shanghai, China. The α diversity index and β diversity index were generated by Pasono Biotech (Shanghai, China) [18].

### 2.10. SCFAs

Following the method outlined by Liu et al. [19], a gram of cecal material was mixed with 15 mL of distilled water and spun at 5000 revolutions per minute for a duration of 10 min. Following this, 1 mL of the supernatant liquid was collected and mixed with 0.2 mL of a 25% metaphosphoric acid solution. Subsequently, the solution was centrifuged at 12,000 rpm for 10 min. The supernatant was gathered and filtered using a 0.22-micron membrane. The concentration of SCFAs in the supernatant was measured using high-performance ion chromatography (Thermo Scientific Dionex Ics-5000+, Waltham, MA, USA).

### 2.11. Statistical Analysis

Statistical analysis was performed using SPSS 23.0 software, with data expressed as the mean ± SEM. All data were assessed for normality and homogeneity of variance. If the assumptions were met, one-way ANOVA was performed, followed by Duncan’s test for comparisons. In cases where the assumptions were not met, non-parametric tests were utilized (Kruskal–Wallis test followed by Dunn’s post hoc test) [20]. Spearman’s correlation analysis was employed to assess the correlation between differential bacterial genera and SCFAs. The data were considered significant at a *p*-value threshold of less than 0.05. Histograms were plotted with GraphPad Prism version 8.

## 3. Results

### 3.1. Effects of Lactic Acid and Glyceryl Lactate on Growth Performance of Piglets

As shown in Table 1, the final body weight and ADG of piglets in both the GL and LG groups were notably greater than those in the control group (*p* < 0.05). Conversely, the GL and LG groups exhibited a notably reduced F:G ratio and diarrhea incidence as opposed to the control group. Furthermore, supplementing the diet with lactic acid notably decreased the diarrhea rate among piglets (*p* < 0.05), yet it did not significantly impact their final body weight, ADG, ADFI, or the F:G ratio.

### 3.2. Effects of Lactic Acid and Glyceryl Lactate on pH and Digestive Enzyme Activities of Digesta of Piglets

**Figure 1:**
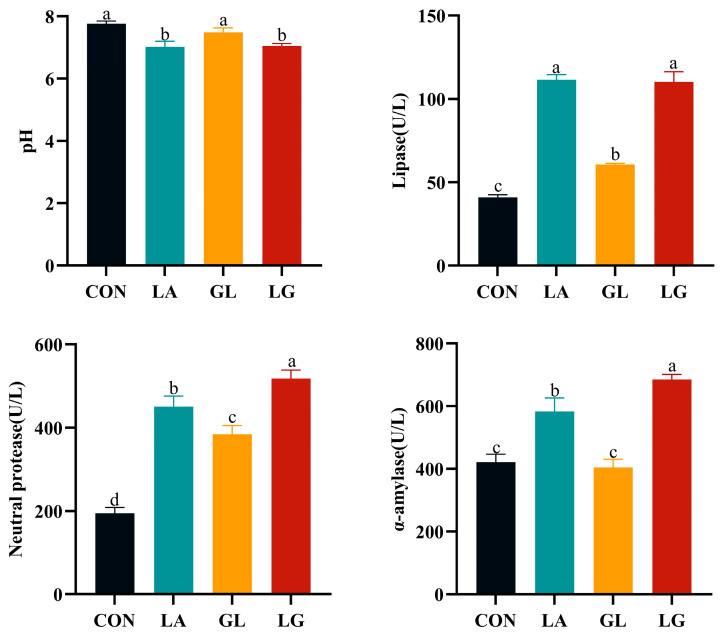
Effects of acidifiers on the pH and digestive enzyme activities of jejunal digesta. Values are mean ± SEM, *n* = 6. ^a^, ^b^, ^c^: means without common letters differ at *p* < 0.05. CON = basal diet; LA = basal diet with 0.5% lactic acid; GL = basal diet with 0.5% glyceryl lactate; LG = basal diet with 0.5% lactic acid and 0.5% glyceryl lactate.

Figure 1 illustrates that the intestinal chyme pH was significantly lower in the LA and LG groups compared to the control group. Concurrently, the enzymatic activities of lipase, neutral protease, and α-amylase were substantially elevated in these groups (*p* < 0.05). Additionally, the incorporation of glyceryl lactate significantly increased the activities of lipase and neutral protease (*p* < 0.05).

### 3.3. Effects of Lactic Acid and Glyceryl Lactate Jejunum Morphology of Piglets

**Figure 2:**
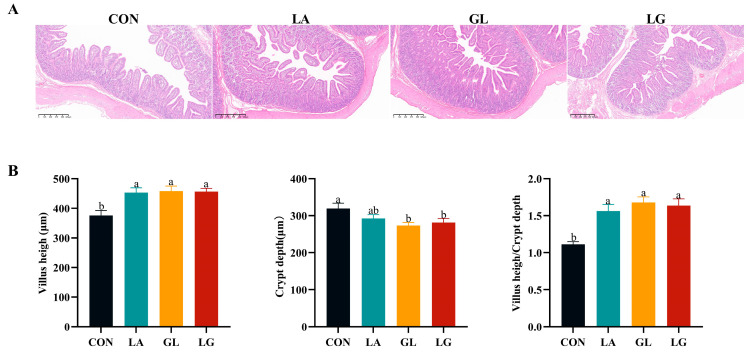
Effects of acidifiers on the morphology and histopathology of the jejunum in weaned piglets. (**A**) Histopathological sections of the jejunum on day 28; (**B**) morphological changes, including villus height, crypt depth, and villus height-to-crypt depth ratio, on day 28. Results are presented as mean ± SEM, *n* = 6. ^a^, ^b^: means without common letters differ at *p* < 0.05. CON = basal diet; LA = basal diet with 0.5% lactic acid; GL = basal diet with 0.5% glyceryl lactate; LG = basal diet with 0.5% lactic acid and 0.5% glyceryl lactate.

Figure 2 shows that histopathological analysis revealed ruptured villi and mild vacuolar degeneration of epithelial cells in both the CON and LA groups. In contrast, no corresponding pathological changes were detected in the GL and LG groups. The LA, LG, and GL groups demonstrated significantly elevated villus crypt ratios when compared to the control group (*p* < 0.05).

### 3.4. Effect of Lactic Acid and Glyceryl Lactate on Jejunal Tight Junction Proteins and Mucins in Piglets

**Figure 3:**
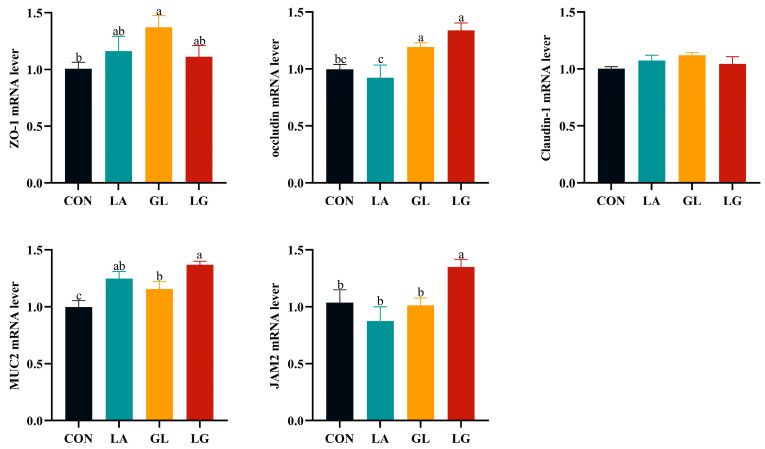
Effects of acidifiers on the relative mRNA abundance of tight junction genes in the jejunum of piglets. Values are expressed as mean ± SEM, *n* = 6. ^a^, ^b^, ^c^: means without common letters differ at *p* < 0.05. CON = basal diet; LA = basal diet with 0.5% lactic acid; GL = basal diet with 0.5% glyceryl lactate; LG = basal diet with 0.5% lactic acid and 0.5% glyceryl lactate.

Figure 3 shows that the inclusion of glyceryl lactate and mixed acids led to a significant increase in the expression of the *occludin* and *MUC2* genes (*p* < 0.05). Furthermore, glyceryl lactate notably stimulated the expression of the *ZO-1* gene, whereas the addition of mixed acids considerably enhanced the expression of the *JAM2* gene (*p* < 0.05).

### 3.5. Effects of Lactic Acid and Glyceryl Lactate Jejunum Antioxidant Markers in Piglets

Table 2 indicates that, across all groups, the jejunum of piglets exhibited significantly elevated levels of GPX and CAT (*p* < 0.05). Moreover, the addition of a mixture of 0.5% lactic acid and 0.5% glyceryl lactate significantly increased jejunal SOD levels in piglets (*p* < 0.05). Simultaneously, the incorporation of lactic acid and mixed acids led to a notable decrease in malondialdehyde (MDA) levels (*p* < 0.05).

### 3.6. Effects of Lactic Acid and Glyceryl Lactate on mRNA Relative Expression of Nrf2 Signaling Pathway in Jejunum of Piglets

**Figure 4:**
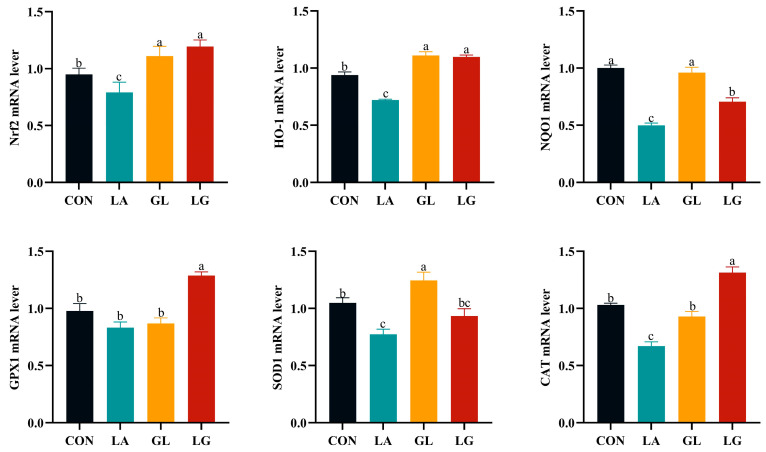
Effects of acidifiers on the relative mRNA abundance of antioxidant genes in the jejunum of weaned piglets. Results are expressed as mean ± SEM. *n* = 6. ^a^, ^b^, ^c^: means without common letters differ at *p* < 0.05. CON = basal diet; LA = basal diet with 0.5% lactic acid; GL = basal diet with 0.5% glyceryl lactate; LG = basal diet with 0.5% lactic acid and 0.5% glyceryl lactate.

Referencing Figure 4, it was evident that the incorporation of glyceryl lactate led to a marked elevation in the relative expression of the genes *Nrf2*, *HO-1*, and *SOD1* (*p* < 0.05). The addition of mixed acids significantly increased the relative expression levels of the *Nrf2*, *HO-1*, *GPX1*, and *CAT* genes (*p* < 0.05).

### 3.7. Effects of Lactic Acid and Glyceryl Lactate on α and β Diversity of Cecum Microorganisms in Piglets

**Figure 5:**
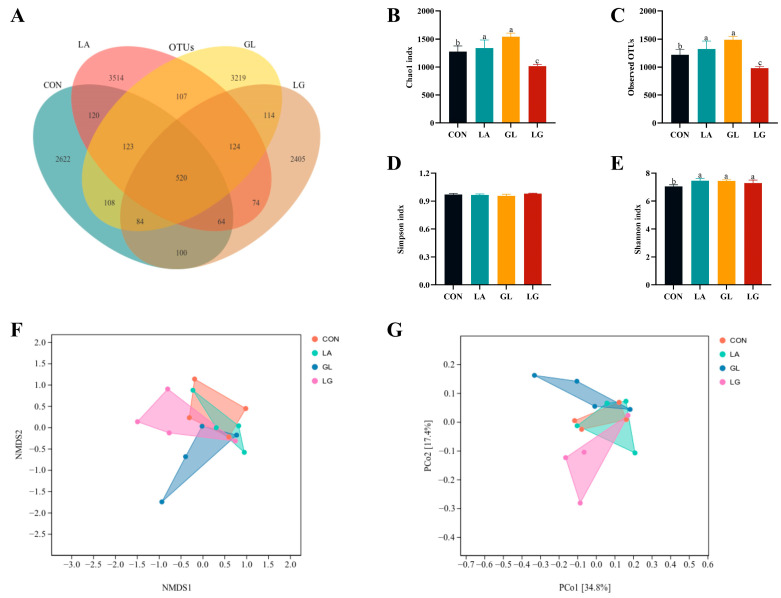
Effects of acidifiers on α and β diversity in the cecum of piglets. (**A**) Venn diagram showing unique and shared ASVs among the four treatment groups. The numbers represent the number of common elements between different sets or the unique elements within a specific set. (**B**–**E**) Comparison of alpha diversity indices, including Chao1 index, observed OTUs, Simpson index, and Shannon index. (**F**,**G**) Weighted UniFrac distance matrix analysis visualized by NMDS and PCoA, where each point represents a sample, and colors indicate different groups. The results are expressed as mean ± SEM. *n* = 4, ^a^, ^b^, ^c^: means without common letters differ at *p* < 0.05. CON = basal diet; LA = basal diet with 0.5% lactic acid; GL = basal diet with 0.5% glyceryl lactate; LG = basal diet with 0.5% lactic acid and 0.5% glyceryl lactate.

Sequencing data revealed a total of 520 shared variant sequences among the four groups, with respective counts of 2622 for the control group, 3514 for the LA group, 3219 for the GL group, and 2405 for the LG group (Figure 5A). The α-diversity index indicated that the addition of glyceryl lactate and mixed acids significantly elevated the Chao1 index and the number of observed OTUs (*p* < 0.05). Although the Simpson index did not exhibit significant variation, the Shannon index showed a significant increase in the three experimental groups (*p* < 0.05) (Figure 5B–E). Principal coordinate analysis showed that the addition of glycerol lactate and mixed acids significantly altered the cecum microbiota (*p* < 0.05) (Figure 5F,G).

### 3.8. Effects of Lactic Acid and Glyceryl Lactate on Phylum Level and Genus Level in Cecum of Piglets

**Figure 6:**
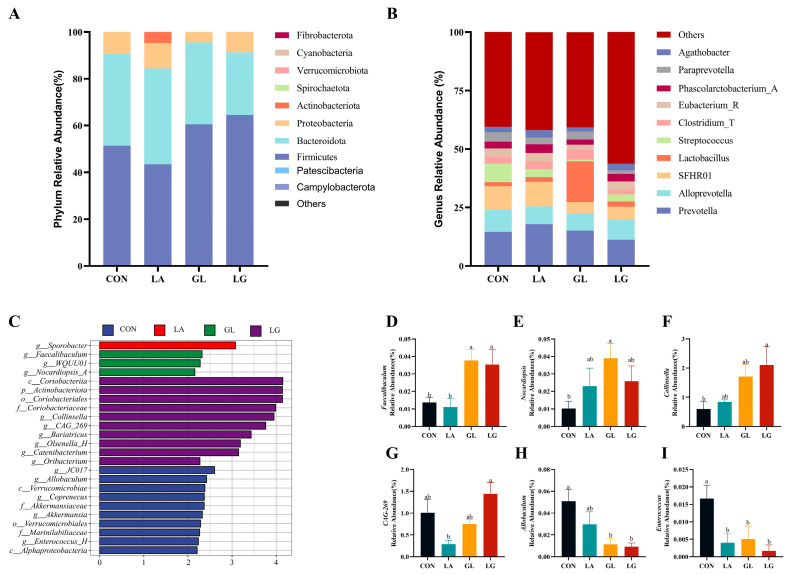
Composition of the microbial community at the phylum and genus levels. (**A**,**B**) Colored bar charts show the relative abundance of microbial taxa. (**C**) LEfSe analysis identifies differentially abundant taxa in the cecal chyme microbiota community, showing only taxonomies with LDA scores > 2. (**D**–**I**) Relative abundances of *Faecalibaculum*, *Nocardiopsis*, *Collinsella*, *CAG269*, *Allobaculum*, and *Enterococcus* across the four groups. Results are expressed as mean ± SEM, *n* = 4. ^a^, ^b^: means without common letters differ at *p* < 0.05. CON = basal diet; LA = basal diet with 0.5% lactic acid; GL = basal diet with 0.5% glyceryl lactate; LG = basal diet with 0.5% lactic acid and 0.5% glyceryl lactate.

At the phylum level, the cecum was predominantly inhabited by Firmicutes, Bacteroidetes, Proteobacteria, Actinobacteria, and Spirochaetota (Figure 6A). At the genus level, the seven predominant bacterial genera identified in the cecal microbiota included *Prevotella*, *Alloprevotella*, *SFHR01*, *Lactobacillus*, *Streptococcus*, *Eubacterium*, and *Phascolarctobacterium*, which likely play a crucial role in cecal function (Figure 6B). Analysis utilizing linear discriminant analysis effect size (LDA) scores greater than 2, as determined by LEfSe analysis, identified distinct microbial communities among the four groups. A total of 24 distinct microbial taxa were observed throughout the study, distributed as follows: 10 in the control group, 1 in the lactic acid-treated group, 3 in the glyceryl lactate group, and 10 in the mixed-acids group (Figure 6C). The incorporation of glycerol lactate notably enhanced the comparative prevalence of *Faecalibaculum* and *Nocardiopsis*; the addition of mixed acids significantly elevated the relative abundance of *Faecalibaculum*, *Collinsella*, and *CAG269* (*p* < 0.05). Meanwhile, *Allobaculum* and *Enterococcus* in both the GL and LG groups were significantly decreased (*p* < 0.05) (Figure 6D–I).

### 3.9. Effects of Lactic Acid and Glyceryl Lactate on the Concentration of SCFAs in the Cecum of Piglets

Table 3 shows that the levels of acetic acid in the three experimental groups were significantly higher than those in the control group (*p* < 0.05). The addition of 0.5% glyceryl lactate alone and a mixture of 0.5% lactic acid with 0.5% glyceryl lactate both significantly increased the butyric acid content (*p* < 0.05).

### 3.10. Correlation Analysis Between Differential Bacteria and SCFAs

**Figure 7:**
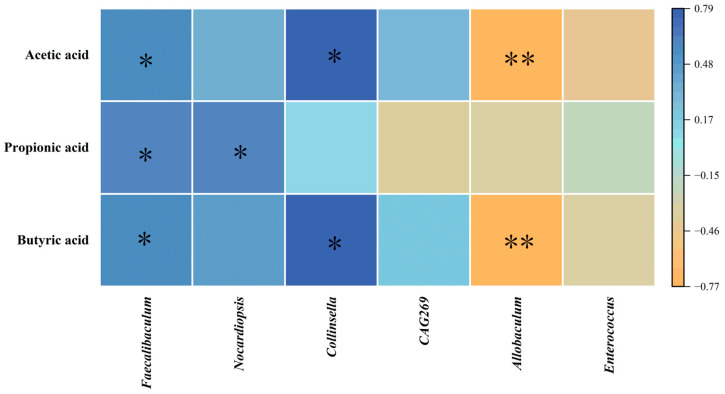
Spearman correlation analysis between differential bacteria and SCFAs. * *p* < 0.05, ** *p* < 0.01.

Spearman correlation analysis was conducted to assess the associations between differential bacteria and SCFAs (Figure 7). The results indicated that *Faecalibaculum* exhibited positive associations with acetic, propionic, and butyric acids. Furthermore, *Nocardiopsis* demonstrated a positive correlation with propionic acid. *Collinsella* was positively correlated with acetic and butyric acids, whereas *Allobaculum* displayed negative correlations with both acetic and butyric acids.

## 4. Discussion

When piglets are weaned, dietary and environmental changes can induce weaning stress. The transition from easily digestible liquid milk to more complex solid diets, which are more difficult to digest, places significant stress on the gastrointestinal system. This often results in digestive issues and diarrhea, which adversely affects growth and performance [21]. This research has shown that 0.5% glyceryl lactate alone and a mixture of 0.5% lactic acid with 0.5% glyceryl lactate significantly enhance the growth performance of piglets, while both supplements and the mixture effectively reduce the incidence of diarrhea. Furthermore, the research of Xu et al. [22] demonstrated that the addition of acidifiers to drinking water led to enhanced growth performance and survival rates in weaned piglets. Additionally, Li et al. [23] found that incorporating 2000 mg/kg of a complex organic acid mixture—comprised of at least 23.9% formic acid, 14.5% lactic acid, and 4.0% citric acid—into the diets of piglets significantly improved daily weight gain and reduced the frequency of diarrhea. Research has consistently indicated that organic acids, whether utilized individually or in combinations, can enhance growth and health outcomes in young pigs [24], broiler chickens [25], and quail [26]. Consistent with our study, pH is a critical factor in an animal’s digestive environment and directly influences digestion and nutrient absorption. The gastrointestinal systems of recently weaned piglets are immature, characterized by weak gastric acid secretion, low lactic acid production, and a gastrointestinal pH that is highly susceptible to external influences. Full-priced feeds contain many acid-buffering substances, such as calcium hydrogen phosphate and stone powder, which can raise intestinal pH compared to levels prior to weaning. An increase in pH can inhibit the function of digestive enzymes and alter the intestinal microbiota, thereby compromising the digestive environment, reducing the surface area available for digestion and absorption, causing damage to the intestinal mucosal barrier, and lowering immunity [27]. In this experiment, we observed that both 0.5% lactic acid and a mixture of 0.5% lactic acid and 0.5% glyceryl lactate significantly lowered intestinal cecal pH while enhancing digestive enzyme activity. The study found that incorporating organic acids in feed reduces gastrointestinal pH, curbs the proliferation of harmful bacteria, and increases protease activity, thereby facilitating nutrient digestion [7]. Hoseini et al. [28] further disclosed that dietary supplementation with lactic acid boosted the intestinal enzymatic activities of trypsin, lipase, and amylase in carp. In summary, the addition of acidifiers to diets not only boosts piglet growth performance but also maintains optimal intestinal pH, positioning lactic acid and glyceryl lactate as promising options for improving piglet growth performance.

The dimensions of intestinal villi and crypts, as well as their ratio, are crucial for assessing the health and functionality of the intestines [29]. Weaning piglets at an early age leads to a reduction in the size of intestinal villi and an extension of crypts. This results in a diminished absorptive surface area within the small intestine, consequently lowering the efficiency of nutrient absorption [30]. The intestinal structure of weaned piglets was found to be enhanced by 0.5% lactic acid, 0.5% glyceryl lactate, and a mixture of 0.5% lactic acid and 0.5% glyceryl lactate, according to the study’s results. Abd El-Ghany [31] demonstrated that dietary supplementation with 0.5% and 1.0% formic acid significantly increased intestinal villus height. Additionally, acidifier supplementation has been shown to enhance gut morphology in Coturnix japonica [26], which aligns with our findings. SCFAs enhance the integrity of the intestinal mechanical barrier by strengthening the tight junctions between intestinal epithelial cells [32]. Proteins forming tight junctions are essential for the physical barrier of the intestines. This barrier, predominantly composed of membrane protein complexes such as junctional adhesion molecules (JAM), occludin, and claudins, acts as the first line of defense against harmful substances [33]. Modifications to the structure and function of tight junctions can potentially undermine the completeness of the intestinal barrier. Mucins (MUC) are key components of the intestinal mucosal barrier, secreted by goblet cells, forming the initial defense within the mucosal barrier, inhibiting pathogen adhesion, and preserving the intestinal environment’s homeostasis [34]. In our experiment, we observed that both 0.5% glyceryl lactate and a mixture of 0.5% lactic acid and 0.5% glyceryl lactate significantly increased the relative gene expression of the tight junction protein *occludin*, as well as the mucin *MUC2*. Research by Ma et al. [35] indicated that feeding with microencapsulated essential oils and organic acids led to an upregulation of mRNA levels for *occludin*, *claudin-1*, and *MUC2* in the ileum of piglets. These findings suggest that incorporating an acidifier can enhance both the morphological structure and the barrier functionality of the intestines in piglets.

The practice of early weaning in animal production has been observed to induce a significant increase in reactive oxygen species (ROS) production in piglets, which has been identified as a primary contributor to induced oxidative stress [36]. Antioxidant enzyme levels, along with MDA and hydrogen peroxide (H_2_O_2_), are commonly used indicators to evaluate the state of oxidative stress within the body [37]. Research findings indicate significant increases in the levels of antioxidant enzymes such as GPX and CAT, coupled with a notable decrease in MDA levels within the GL and LG groups. Additionally, Xu et al. [22] found that the administration of acidifiers in drinking water enhanced serum catalase levels and T-AOC while concurrently reducing MDA concentrations in weaned piglets. These results align with our findings. SCFAs exhibit antioxidant effects as modulators of *Nrf2* redox signaling, with many studies focusing primarily on butyrate [38]. A wealth of research has demonstrated that antioxidant enzymes, including SOD and GSH, are under the regulatory control of the *Nrf2* signaling pathway. The study showed that acidifying agents protect cells from ROS damage by activating the *Nrf2* signaling pathway, increasing SOD and GSH enzyme activities and decreasing MDA levels [39,40]. In our experiment, we observed a significant increase in the relative expression of the *Nrf2* and *HO-1* genes in the *Nrf2* signaling pathway among the GL and LG groups, as well as elevated levels of *SOD1* in the GL group and the *GPX1* and *CAT* genes in the LG group. Chen et al. [41] reported that chlorogenic acid enhances the antioxidant defenses in piglets by inhibiting the *TLR4*/*NF-κB* pathway and activating the *Nrf2* pathway, which leads to increased levels of jejunal glutathione peroxidase and catalase, alongside reduced levels of MDA. Moreover, Xiao et al. [42] documented that the incorporation of ellagic acid in the diet enhanced the expression of *Nrf2* protein, which in turn increased the concentrations of *HO-1* and *NQO1* proteins in the mucosa of the small intestine. In summary, lactic acid and glyceryl lactate enhance intestinal oxidative stress levels, potentially through the activation of the *Nrf2*/*HO-1* signaling pathway.

Gut microbes also contribute actively to the preservation of gut health in weaned piglets [43]. SCFAs are the end products of microbial fermentation of carbohydrates, and fermentation occurs primarily in the hindgut of the animal intestine. Acetate, propionate, and butyrate constitute the primary SCFAs, with their molar proportions being 12:5:3 [7]. No single bacterium is capable of breaking down all nutrient substrates or producing all three SCFAs via carbohydrate fermentation. Consequently, the diversity and distribution of SCFAs in the gut reflect metabolic collaboration among various microorganisms [32]. In this study, *Faecalibaculum* demonstrated a significant increase, whereas *Allobaculum* and *Enterococcus* showed significant decreases in the GL and LG groups. Additionally, there was a notable increase in *Nocardiopsis* in the GL group and in *Collinsella* and *CAG269* in the LG group. *Faecalibaculum* is recognized for its ability to produce butyrate, contributing to the regulation of homeostasis in intestinal epithelial cells [44]. *Collinsella* has antioxidant and anti-apoptotic effects [45]. Although *Collinsella* is not a major SCFA-producing bacterium, it has been suggested that *Collinsella* may be indirectly involved in regulating the production of SCFAs by influencing the endocannabinoid system [46]. Thus, the elevation of butyric acid levels in the GL and LG groups may be attributed to the increased presence of both *Faecalibaculum* and *Collinsella*, facilitating reduced jejunal oxidative stress through the Nrf2 signaling pathway. Although *Allobaculum* and *Enterococcus* are known to generate SCFAs and support intestinal barrier function, their levels were reduced in the GL and LG groups. This reduction may stem from the intestinal microbiota’s limited capacity to efficiently metabolize these organic acids, leading to slower growth rates for these genera [47]. Furthermore, *Nocardiopsis* was capable of producing a variety of active substances, such as surfactant, anti-cancer substances, etc. [48], and *CAG269* has been associated with fat accumulation in the liver [49]. This study did not elucidate the reasons behind the observed changes in the populations of these two genera within the gut, warranting further investigation to understand their potential implications for intestinal health.

## 5. Conclusions

In summary, our findings indicate that the addition of 0.5% glyceryl lactate, either alone or in combination with 0.5% lactic acid, positively influences the growth performance and intestinal health of weaned piglets. The mechanisms underlying these benefits may be associated with enhanced intestinal antioxidant capacity, alterations in gut microbiota composition, and increased SCFA levels. 

## Figures and Tables

**Table 1:** Effects of lactic acid and glyceryl lactate on the growth performance of piglets.

Contents	CON	LA	GL	LG	SEM	*p*-Value
Initial weight, kg/piglet	7.99	7.89	7.92	7.89	0.03	0.794
Final weight, kg/piglet	19.41 ^c^	19.93 ^bc^	21.30 ^ab^	21.89 ^a^	0.32	0.010
ADG, kg/d	0.41 ^c^	0.43 ^bc^	0.48 ^ab^	0.50 ^a^	0.01	0.023
ADFI, kg/d	0.69	0.68	0.74	0.75	0.01	0.113
F/G, kg/kg	1.70 ^a^	1.59 ^ab^	1.57 ^b^	1.49 ^b^	0.02	0.020
Diarrhea rate, %	7.96 ^a^	3.56 ^b^	3.27 ^b^	3.29 ^b^	0.67	0.020

Values are mean ± SEM, *n* = 6. ^a^, ^b^, ^c^: means within a row with no common superscripts differ significantly, *p* < 0.05. CON = basal diet; LA = basal diet with 0.5% lactic acid; GL = basal diet with 0.5% glyceryl lactate; LG = basal diet with 0.5% lactic acid and 0.5% glyceryl lactate.

**Table 2:** Effects of lactic acid and glyceryl lactate jejunum antioxidant markers in piglets.

Contents	CON	LA	GL	LG	SEM	*p*-Value
GPX, U/mL	55.40 ^c^	94.48 ^b^	119.65 ^a^	136.58 ^a^	7.02	0.013
CAT, U/mL	36.39 ^c^	45.13 ^b^	54.30 ^a^	55.04 ^a^	2.03	0.016
SOD, U/mL	90.27 ^bc^	81.28 ^c^	95.73 ^b^	109.93 ^a^	2.87	0.014
T-AOC, U/L	1.15	1.26	1.38	1.40	0.03	0.060
MDA, nmol/mL	1.83 ^a^	1.65 ^b^	1.56 ^b^	1.48 ^b^	0.04	0.020
GPX, U/mL	55.40 ^c^	94.48 ^b^	119.65 ^a^	136.58 ^a^	7.02	0.013

Values are mean ± SEM, *n* = 6. ^a^, ^b^, ^c^: means within a row with no common superscripts differ significantly, *p* < 0.05. CON = basal diet; LA = basal diet with 0.5% lactic acid; GL = basal diet with 0.5% glyceryl lactate; LG = basal diet with 0.5% lactic acid and 0.5% glyceryl lactate.

**Table 3:** Effects of lactic acid and glyceryl lactate on the concentration of SCFAs in the cecum of piglets.

Contents	CON	LA	GL	LG	SEM	*p*-Value
Acetic acid, mg/g	2.14 ^b^	3.04 ^a^	2.87 ^a^	2.81 ^a^	0.11	0.005
Propionic acid, mg/g	0.92 ^b^	1.23 ^a^	1.19 ^a^	1.08 ^ab^	0.04	0.013
Butyric acid, mg/g	0.31 ^c^	0.38 ^c^	0.53 ^b^	0.62 ^a^	0.03	0.001
Isovaleric acid, mg/g	0.02	0.04	0.06	0.1	0.01	0.098
Valeric acid, mg/g	0.12	0.21	0.29	0.21	0.03	0.375

Values are mean ± SEM, *n* = 6. ^a^, ^b^, ^c^: means within a row with no common superscripts differ significantly at *p* < 0.05. CON = basal diet; LA = basal diet with 0.5% lactic acid; GL = basal diet with 0.5% glyceryl lactate; LG = basal diet with 0.5% lactic acid and 0.5% glyceryl lactate.

## Data Availability

Data are contained within the article.

## Supplementary Materials

The following supporting information can be downloaded at: https://www.mdpi.com/article/10.3390/antiox14040391/s1, Table S1: Basal diet composition and nutrient level; Table S2: Diarrhea index score; Table S3: List of primers used for Q-PCR analysis.
