# Supplementary material for: Effects of Lactic Acid and Glyceryl Lactate on Growth Performance, Antioxidant Capacity, and Intestinal Health of Piglets
Source: Antioxidants (Basel). 2025 Mar 26;14(4):391. doi: 10.3390/antiox14040391 (PMC12023924; doi:10.3390/antiox14040391)
Supplement: Supplementary file 1 [file antioxidants-14-00391-s001.zip › antioxidants-3512433-supplementary.pdf]

Table S1. Basic diet composition and nutrient composition

Item	Contents
Corn	42.05
Wheat	20.00
Corn germ meal	3.00
Soybean meal	4.30
Soy protein concentrate	7.08
Extruded soybean	10.00
Fish meal	1.00
Whey powder	2.00
Glucose	2.80
Soybean oil	3.00
Stone powder	1.15
Calcium dihydrogen phosphate	0.85
Sodium chloride	0.20
Choline chloride	0.08
L-lysine hydrochloride (98.5%)	0.65
DL-methionine	0.11
L-threonine	0.20
L-tryptophan	0.03
Premix[1]	1.50
**Calculated nutrient levels**	
ME (MJ/kg)	14.68
CP %	18.59
Ca %	0.70
Total phosphorus %	0.61
Available phosphorus %	0.36
Lys %	1.40
Met %	0.40
Thr %	0.87
**Analyzed nutrient levels**	
Gross energy (MJ/kg)	16.19
CP %	17.86
Ca %	0.66
Lys %	1.38
Met %	0.42
Thr %	0.89

[1] Provided per kilogram of diet: VA 9510 IU, VD3 3290 IU, VE 50IU, VK3 4.50 mg, VB1 3.50mg, VB2 6.70 mg, vitamin B6 3.00 mg, vitamin B12 0.03 mg, folic acid 1.20 mg, niacin 32.50 mg, pantothenic acid 28.00 mg, biotin 0.20 mg, choline chloride

1000.00 mg, iron 210.00 mg, copper 124.00 mg, zinc 1600.00 mg, manganese 58.00 mg, iodine 1.30 mg, selenium 0.45 mg

Table S2. Diarrhea index score.

Degree of diarrhea	Appearance of stool	Diarrhea score
Normal	Hard bars or grainy	0
Mild	Soft stool, can form	1
Moderate	Mushy, unformed	2
Severe	Liquid, fecal–water separation	3

Table S3. Primer sequence of target and reference genes.

Gene[1]	GenBank number	primer (5'-3')[2]	Product size(bp)
Claudin-1	NM_001244539.1	F: GCCACTGTTGGCATGAAGTG<br>R: CTGGCATTGACTGGGGTCAT	182
Occludin	NM_001163647.2	F: CAGGTGCACCCTCCAGATTG<br>R: ATGTCGTTGCTGGGTGCATA	167
ZO-1	XM_021098860.1	F: GCCAGCTGGAGCTTAGAACA<br>R: GGCATCAAGAGGGGCTACTG	152
MUC2	XM_021082584.1	F: CTGTGCGACTACAACTTCGC<br>R: AGATGGTGTCGTCCTTGACC	139
JAM2	XM_021070861.1	F: GATGCCCAAAGCACCAACAG<br>R: TGTCCGACAGAATTACGGGC	122
Nrf2	XM_021075130.1	F: GAAAGCCCAGTCTTCATTGC<br>R: TTGGAACCGTGCTAGTCTCA	190
HO-1	NM_001004027.1	F: TGTAGACCGGGTTCTCCTTG<br>R: GCTGAGAATGCCGAGTTCAT	142
NQO1	NM_001159613.1	F: TGAATTACATCTCTGTGGTTTA<br>R: AGAATGACACTCATATTAGGCG	171
GPX1	NM_214201.1	F: CTGGTCGTGCTCGGCTTCC<br>R: GCCTGGTCGGACGTACTTGAG	96
SOD1	NM_001190422.1	F: GATCAAGAGAGGCACGTTGGA<br>R: GTGGCCACACCATCTTTGC	62
CAT	XM_021081498.1	F: CATTGAGCCCAGCCCTGACAAG<br>R: AGGCGGTGGCGGTGAGTG	72
β-Actin	XM_003124280.5	F: CTGCGGCATCCACGAAACT<br>R: AGGGCCGTGATCTCCTTCTG	147

[1] *ZO-1* = zonula occludens-1; *MUC2* = mucin 2; *JAM2* = junctional adhesion molecule 2; *Nrf2* = NFE2-like bZIP transcription factor 2; *HO-1* = heme oxygenase 1; *NQO1* = NAD(P)H quinone dehydrogenase 1; *GPX1* = glutathione peroxidase 1; *SOD1* = superoxide dismutase 1; *CAT* = catalase; *β-Actin*= beta-actin

[2] F, Forward; R, Reverse.
